# Dietary Supplements: Which Place between Food and Drugs?

**DOI:** 10.3390/nu12010204

**Published:** 2020-01-13

**Authors:** Catherine Féart

**Affiliations:** Univ. Bordeaux, Inserm, BPH, Team LEHA, UMR 1219, F-33000 Bordeaux, France; catherine.feart-couret@u-bordeaux.fr; Tel.: +33-547304204

Healthy dietary habits and food choices, a part of lifestyle, are recognized as major environmental factors for the prevention of non-communicable chronic diseases over the life course; their modifiable features promise a reduced socio-economic global burden load in aging societies [1]. To better help satisfy nutritional needs, the use of dietary supplements has also become a common practice. Initially, this practice was acknowledged to avoid deficiencies in some essential micronutrients and their adverse health consequences, and its use was not questioned. This practice seems now to have acquired the role of comforting consumers facing an ever increasing and nutritionally inadequate food supply. Therefore, dietary supplements use is now a form of food behavior, and consumers, manufacturers, and regulatory officers have to consider and evaluate their safety, efficacy, and quality. However, the concerned parties appear to assign different priorities to these three requirements, according to their needs and expectations. To complicate the picture further, the definition of dietary supplements has not yet reached a consensus worldwide. These issues have been extremely well documented by Dwyer and colleagues in a recent review published in *Nutrients* [2]. This review comprehensively explained the major challenges in the field of dietary supplements, from the need for a standardized definition to the evolution of quality assessment in time and different countries, the existing legislation that should be applied for groups of molecules or single molecules, the evolving regulatory landscape, the legitimate expectations of consumers about safety, quality, and efficacy.

Therefore, the above-mentioned authors acknowledged that there is a need for consensual regulatory rules over the world, but this cannot be reached as long as a consensual definition of dietary supplements is missing: natural health products, complementary medicines, food supplements, phytomedicine are all relevant terms used for dietary supplements, sometimes restricted to specific cultural contexts. Moreover, various legal systems, levels of economic development, and regulations applied to dietary supplements limit the generalization of standardized rules. For instance, the application of rules already in place for the development of a therapeutic drug to the development of a dietary supplement would be inconceivable in most countries. In fact, the expected benefits of a dietary supplement are not as significant as those of a drug, and the target population may be different (e.g., healthy or unhealthy) [3]. Moreover, dietary supplements are consumed as an addition to spontaneous food intake, and therefore, their baseline level is usually far from zero, in contrast to that of an active ingredient of a newly developed drug. To go a step further, it should be mentioned that the matrix effect of dietary components is also a major condition for the efficacy of a product. Overall, it appears that the gold standard design applied for the development of therapeutic drugs, i.e., the randomized controlled trial, should not be the exclusive one to prove the efficacy of dietary supplements.

Supplements’ quality, safety, and efficacy remain the main issues for each involved party, i.e., the consumers, the manufacturers, and the regulatory officers. The dose of each bioactive supplements and their possible interactions are important to ensure their safety and efficacy. Additionally, technological challenges have still to be overcome to establish certified references materials and standardized operational protocols. The final goal is the definition of biomarkers that could be used as indicators of safety and efficacy, the latter being related to markers of health outcomes and, importantly, to customer expectations. A better understanding of the underlying mechanisms of action of bioactive substances will help identify molecules with preventive or therapeutic activity [4]. To date, the efficacy of most dietary supplements still needs to be demonstrated. Regarding the supplement-producing firms, an accurate quality control of their developed dietary supplements should be the rule in order to gain the confidence of customers, who, by choosing a reliable dietary supplement, will in turn support the manufacturers. This will also allow claims to be established. Finally, the profile of dietary supplements’ users has to be better defined; in this respect, the role of pharmacists in the education of dietary supplements’ users remains a major challenge [5]. The future will have to take into account both personalized nutrition and personalized medicine based on the genetic and microbiota susceptibility of the consumers. This last challenge will require increased international collaborations among scientists with complementary skills to ensure the best quality of life as possible for dietary supplements’ consumers.
